# Oral iron acutely elevates bacterial growth in human serum

**DOI:** 10.1038/srep16670

**Published:** 2015-11-23

**Authors:** James H. Cross, Richard S. Bradbury, Anthony J. Fulford, Amadou T. Jallow, Rita Wegmüller, Andrew M. Prentice, Carla Cerami

**Affiliations:** 1 MRC Keneba, MRC Unit, The Gambia, Atlantic Blvd, Serrekunda, Gambia; 2 School of Medical and Applied Sciences, Central Queensland University, North Rockhampton, Queensland, Australia; 3 MRC International Nutrition Group, London School of Hygiene & Tropical Medicine, Keppel Street, WC1E 7HT, London, UK; 4 Department of Epidemiology, Gillings’ School of Global Public Health, University of North Carolina, Chapel Hill, North Carolina, USA

## Abstract

Iron deficiency is the most common nutrient deficiency worldwide and routine supplementation is standard policy for pregnant mothers and children in most low-income countries. However, iron lies at the center of host-pathogen competition for nutritional resources and recent trials of iron administration in African and Asian children have resulted in significant excesses of serious adverse events including hospitalizations and deaths. Increased rates of malaria, respiratory infections, severe diarrhea and febrile illnesses of unknown origin have all been reported, but the mechanisms are unclear. We here investigated the *ex vivo* growth characteristics of exemplar sentinel bacteria in adult sera collected before and 4 h after oral supplementation with 2 mg/kg iron as ferrous sulfate. *Escherichia coli, Yersinia enterocolitica* and *Salmonella enterica* serovar Typhimurium (all gram-negative bacteria) and *Staphylococcus epidermidis* (gram-positive) showed markedly elevated growth in serum collected after iron supplementation. Growth rates were very strongly correlated with transferrin saturation (p < 0.0001 in all cases). Growth of *Staphylococcus aureus*, which preferentially scavenges heme iron, was unaffected. These data suggest that even modest oral supplements with highly soluble (non-physiological) iron, as typically used in low-income settings, could promote bacteremia by accelerating early phase bacterial growth prior to the induction of immune defenses.

Iron deficiency (ID) remains the most pervasive nutritional deficiency worldwide. The prevalence of ID in mothers and young children frequently exceeds 50% in low-income countries. Insufficient iron impairs growth and cognitive development in childhood^1^,^2^.

Low cost iron supplements are effective for the treatment of ID and in countries with ID rates of >40%, the World Health Organization recommends universal iron supplementation of pregnant women and young children^3^,^4^,^5^. To overcome perceived limitations in the ability to absorb iron, supplements usually employ highly soluble forms of iron (ferrous sulfate or fumarate) given in rather large non-physiological bolus doses. The wisdom of these policies has long been questioned^6^,^7^ and has come under serious scrutiny starting in 2006 with the premature termination of a large trial in Pemba, Tanzania after significant increases in serious adverse outcomes (hospitalizations and deaths) in young children receiving iron-folate supplements were seen^8^. The emphasis was originally focused on malaria as the causative agent for the increases in morbidity and mortality during iron supplementation^9^ but subsequent trials have described excesses of other infections in groups randomized to iron or multiple micronutrients containing iron^10^,^11^,^12^,^13^,^14^,^15^. These findings have paralyzed iron supplementation policies.

The underlying mechanisms and the types of organisms responsible for these clinical and epidemiological observations remain unclear. Recent field studies suggest that oral iron supplementation in children increases susceptibility to bacterial infections, particularly diarrhea^13^,^16^, alters the gut microbiota^16^,^17^, and increases the virulence of many common bacterial enteropathogens^18^,^19^,^20^.

Numerous animal studies over many decades have shown that administration of iron in diverse forms accelerates the growth of peritoneally-injected pathogens, causing a septicemia with rapidly fatal outcomes^21^,^22^. We here examine the possibility in humans that a simple oral dose of supplemental iron could promote bacterial growth in serum. We used a series of e*x vivo* bacterial growth assays with sentinel organisms that were selected on the basis of their varying modes of pathogenesis and abilities to scavenge iron from the host.

## Results

### Oral iron supplementation increases iron parameters in serum

To determine the effects of oral iron supplementation on bacterial growth in human serum, we enrolled 48 normal healthy non-anemic male subjects [mean ± SD: Hemoglobin (Hgb) = 14.5 ± 1.13 g/dL; Mean Corpuscular Volume (MCV) = 83.8 ± 5.5 fL; Ferritin = 62.8 ± 53.2 ng/mL]. Volunteers donated serum immediately before, and then four hours after, oral ingestion of 400 mg ferrous sulfate (containing the equivalent of 130 mg of elemental iron). Transferrin saturation (TSAT) increased from 42.1% (±12.5%, SD) to 75.7% (±18.1%, SD) and total serum iron increased from 30.3 μmol/L (± 10.2μmol/L, SD) to 53.0μmol/L (± 15.8μmol/L, SD) four hours after iron supplementation.

### Effects of oral iron supplementation on *ex vivo* bacterial growth in serum

We next measured the growth of the five species of sentinel bacteria in the baseline and post-dose sera. To account for the between-subject variance in the starting transferrin saturation levels, we used mixed statistical models to allow two nested higher levels of variation: patient and bleed (pre- and post-iron supplementation). This enabled us to independently analyze the effects of TSAT and iron supplementation.

**Figure 1:**
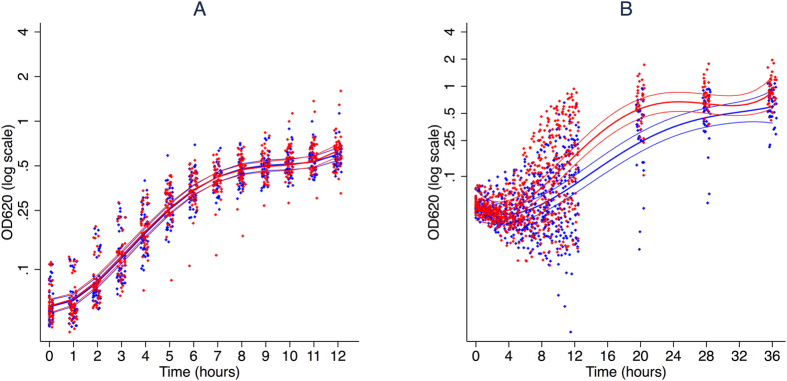
Growth of sentinel gram-positive bacteria in human serum before and after oral iron supplementation. *S. aureus* (**A**) and *S. epidermidis* (**B**) were grown in serum from subjects before (blue) and after (red) oral iron supplementation with 400 mg ferrous sulfate (containing the equivalent of 130 mg of elemental iron). The thicker central lines represent the fitted curves for an average individual and the thinner lines the 95% confidence intervals for these estimates. The points show individual values of OD_620_. Curves were derived from mixed effects models fitting degree-four orthogonal polynomials in time to log(OD_620_), where OD_620_ is the mean optical density of the three replicates at time (t).

*S. aureus*, an organism with a strong preference for heme-derived iron^23^, behaved differently from the other four bacteria (Fig. 1A). Iron supplementation had no impact on the general pattern of the growth curve (p = 0.3). Both the time to reach peak doubling time (p = 0.21), and the doubling time during the exponential growth phase (p = 0.78) were also unchanged by iron supplementation (Table 1). Growth did not correlate with TSAT (p = 0.08) (Table 2).

*S. epidermidis* (Fig. 1B) demonstrated an initial delay in growth in comparison with the other species. Iron supplementation influenced the overall pattern of the growth curve (p < 0.0001). Specifically, iron supplementation reduced the lag phase, the time to reach peak doubling time (p = 0.001), and increased the doubling time during the exponential growth phase (p < 0.001) (Table 1). The very strong effect of iron on the overall increase in bacterial growth (X^2^ = 55 (approximately), p < 0.0001) was equally explained using pre/post supplementation as a dichotomized variable or by TSAT (Table 2).

**Figure 2:**
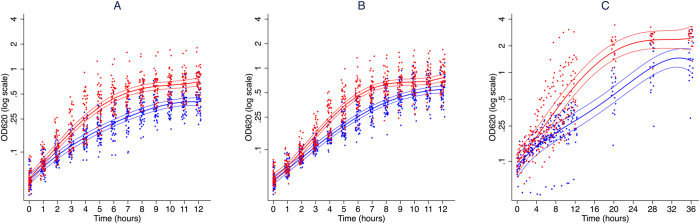
Growth of sentinel gram-negative bacteria in human serum before and after oral iron supplementation. *S.* Typhimurium (**A**), *E. coli* (**B**) and *Y. entercolitica* (**C**) were grown in serum from subjects before (blue) and after (red) oral iron supplementation with 400 mg ferrous sulfate (containing the equivalent of 130 mg of elemental iron). The thicker central lines represent the fitted curves for an average individual and the thinner lines the 95% confidence intervals for these estimates. The points show individual values of OD_620_. Curves were derived from mixed effects models fitting degree-four orthogonal polynomials in time to log(OD_620_), where OD_620_ is the mean optical density of the three replicates at time (t).

*S.* Typhimurium (Fig. 2A) and *E. coli* (Fig. 2B) both showed highly significant differences in their growth curves after iron supplementation (p < 0.0001). The doubling times post-iron supplementation were significantly shorter than pre-iron supplementation (p < 0.0001), but time to reach peak doubling time was unaffected by treatment (Table 1). Transferrin saturation had a very strong effect on overall growth rates of both *S.* Typhimurium (X^2^ = 348, p < 0.0001) and *E. coli* (X^2^ = 300, p < 0.0001), comparable to, indeed a little larger than, that of iron supplementation in both *S.* Typhimurium (X^2^ = 213, p < 0.0001) and *E. coli* (X^2^ = 221, p < 0.0001). For *E. coli*, iron supplementation (X^2^ = 35, p < 0.0001) and TSAT (X^2^ = 69, p < 0.0001) each had significant effects on bacterial growth after controlling for the other. The same held true for *S.* Typhimurium growth where both iron supplementation (X^2^ = 22, p = 0.0004) and TSAT (X^2^ = 105, p < 0.0001) each had significant effects on bacterial growth after controlling for the other (Table 2). This is likely to be because, in addition to capturing the effect of supplementation, transferrin saturation also explains differences between individuals.

For *Y. enterocolitica* (Fig. 2C), the maximum growth rate was difficult to locate. In fact, although the growth curves clearly differ significantly (p < 0.0001), the doubling times at one hour did not differ significantly. As was the case for *S.* Typhimurium and *E. coli*, TSAT had an impact on growth (X^2^ = 120, p < 0.0001) as did iron supplementation (X^2^ = 108, p < 0.0001). Additionally, iron supplementation (X^2^ = 33, p < 0.0001) and TSAT (X^2^ = 37, p < 0.0001) each had significant effects on bacterial growth after controlling for the other (Table 2).

## Discussion

The biologically useful redox characteristics of the Fe(II) to Fe(III) transition place iron apart from other nutrients. Additionally, it lies at the epicenter of the host-pathogen battle for resource control. Host defense mechanisms to withhold iron from invading pathogens are some of the most evolutionarily conserved innate strategies against infection^24^, but most bacterial species have evolved counter-acting strategies for pirating host iron including: (1) receptors that bind transferrin, lactoferrin or hemoglobin; and (2) low molecular weight siderophores that acquire iron from host proteins or from low molecular weight iron compounds^25^.

The potential health threat posed by exogenous iron, repeatedly demonstrated in animal models^25^, has tended to be overlooked in clinical settings. The recent iron trials with adverse outcomes in children in developing countries^8^,^10^,^11^,^12^,^13^,^14^,^15^ have prompted new mechanistic studies providing experimental verification that oral iron adversely modifies the gut microbiome^17^,^26^ and increases the virulence of pathogenic enteric bacteria^18^,^20^. In this study we focused on the issue of systemic, as opposed to enteric, bacterial infections building upon prior knowledge that iron can precipitate septicemias (for instance, based on the disastrous outcomes of intramuscular iron-dextran administration to Polynesian neonates^27^).

The *ex vivo* assays we describe here show that customary oral supplementation with highly-soluble iron as ferrous sulfate can profoundly affect the growth dynamics of four of the five sentinel species we studied. This could potentially undermine a key component of innate immunity allowing such organisms to achieve overwhelming numbers by the time adaptive immune defense mechanisms are up-regulated. Note that the very strong correlations between TSAT and growth rates emphasizes the importance of this variable even in the presence of likely inter-individual differences in other iron-related (e.g. lipocalin-2, haptoglobin) and other (e.g. defensins) non-cellular defense mechanisms within the sera.

Recent molecular insights into human iron metabolism have challenged the basic pillars on which public health strategies involving highly soluble iron supplements have been developed. The prior belief that humans are constitutionally inefficient at absorbing iron, and hence require large non-physiological doses taken apart from food, is now overturned by the knowledge that hepcidin actively down-regulates iron acquisition especially in the presence of an infectious threat^24^. The dual regulation of hepcidin by iron and infection (inflammation) underscores the threat posed by exogenous iron. An increase of hepcidin caused by an infection might have the evolutionary function of decreasing further iron uptake from the intestine to reduce circulating iron fuel for microorganisms. This suggests that we should not interfere via high dose iron supplements.

Transferrin saturation is homeostatically controlled with a normal range between 15–50% in males. Our data show that increasing TSAT from a mean of 42% to 76% profoundly stimulated bacterial growth with a continuous association across all levels of TSAT. The role of TSAT in mediating host susceptibility to infection has been known for almost half a century^28^. However, neither the strength of this association, nor the ability of acute increases in TSAT following iron doses to so rapidly favor bacterial growth, have been previously appreciated. The dose level selected for the adults in this study was based on that most frequently used for young children with iron deficiency (2 mg/kg/day), however our subjects were iron replete. In iron deficient children, hepcidin would be down-regulated to allow maximal iron absorption^29^ and hence TSAT would be expected to rise even further. Although a higher percentage of increase can be expected, the TSAT baseline will be lower and may still end up lower than in iron replete men. In the event of accidental ingress of pathogens through a cut, abrasion or leaky gut, these high levels of TSAT could precipitate a fulminant bacteremia before other cognate immune defenses have time to respond.

Iron absorbed from a natural food matrix, or even when ferrous sulfate is given with food^30^, is released much more slowly and causes minor deviations in TSAT and in non-transferrin bound iron (NTBI)^30^ which, according to the data presented here, would be much less likely to promote bacterial growth. In regions of the world where iron-rich foods are scarce, or too costly, supplementation with slow release nano-molecular formulations that mimic a food matrix^31^ may provide a safer option and might additionally reduce the adverse effects of unabsorbed iron on the gut microbiota. Field trials of such compounds are warranted. Additional safe approaches to supplement iron include the use of oral bovine lactoferrin^32^.

## Materials and Methods

### Subjects

Forty-eight male Gambian subjects (averaging 40y; range 21–64y) were recruited. Subjects had no history of fever, illness or anti-microbial use during the preceding seven days; were malaria rapid test negative and were non-anemic (Hemoglobin >12 g/dL). All patients donated blood between 9 and 10 am, on an empty stomach, immediately prior to and four hours after taking 400 mg ferrous sulfate orally. Food was provided two hours after iron supplementation.

Informed consent was obtained from all subjects. This study was approved by the Gambian Government/MRC Joint Ethics Committee (SCC1312v2) and by the University of North Carolina Institutional Review Board (protocol #143044). All experiments were performed in accordance with the approved guidelines.

### Biochemical Parameters

Complete blood counts were obtained using a Medonic M series (Boule Diagnostics Int AB, Stockholm, Sweden) hematology analyzer. Serum biochemical parameters including serum iron, transferrin, ferritin and transferrin saturation were obtained using a Cobas Integra 400 plus (Roche, Basel, Switzerland) biochemistry analyzer.

### Bacterial Growth assays

*Staphylococcus aureus* (strain NCTC8325), *Staphylococcus epidermidis* (FDA strain *PCI1200*, ATCC12228), *Salmonella enterica serovar* Typhimurium (strain *LT2*, ATCC19585) and *Escherichia coli* (strain *Crooks*, ATCC8739) were grown overnight for 18 hours at 37 °C in 5 mL iron free minimal growth media, Iscove’s Modified Dulbecco’s Medium (IMDM, Invitrogen). This was conducted in air with continuous shaking (250 rpm). A high-virulence, siderophore producing *Yersinia enterocolitica* (strain *WA-314*, ATCC51871) was grown in IMDM containing 10 mM ethylene glycol tetraacetic acid (EGTA, Sigma) (pH7). All growth assays were run in triplicate in IMDM containing 50% heat-inactivated human serum. Bacterial growth was monitored by measuring the optical density at 620 nm (OD_620_) hourly for 12 hours (*Staphylococcus aureus, Salmonella enterica serovar* Typhimurium, and *Escherichia coli)* and then at 20, 28, 36 hours (*Staphylococcus epidermidis* and *Yersinia enterocolitica*) using a Multiscan FC ELISA plate reader (Thermo Scientific).

### Statistical analysis

We analyzed the growth assays with a mixed model using a quartic polynomial in Stata12 (StataCorp, College Station, TX). We used the logarithm of the mean of the three replicate OD readings at each time point as the response variable and modeled growth trajectories by fitting this to orthogonal polynomials (to degree 4) in time using mixed effects models with random intercept and coefficients due to patient and, nested within patient, bleed (pre and post). In effect, therefore, the growth trajectory for the i^th^ blood sample was modeled by an equation of the form:


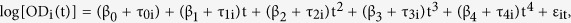


where t is time since inoculation, the βs are estimated coefficients and the τs and ε are the random effects.

For each species of bacterium, we compared its growth pre- and post-iron supplementation by examining differences in (1) Growth Curve i.e. general pattern of the overall growth trajectories; (2) Time to Reach Peak Doubling Time, i.e. the time at which the rate of increase in log[OD] was at a maximum and (iii) Doubling Time During Exponential Growth Phase, i.e. when the doubling time was at its greatest, or, if the lag phase was not detectable at 1 hour after inoculation. We estimated the timing of the maximum slope by setting the second derivative with respect to time of the deterministic component of the model to zero. We calculated the doubling time at time t as log(2)/(slope of log[OD(t)]). We employed the likelihood ratio test to compare models for which the growth patterns were and were not dependent on the transferrin saturation or iron supplementation. We used the delta method to obtain the 95% confidence intervals for the time at which maximum slope occurred and the doubling time.

## Additional Information

**How to cite this article**: Cross, J. H. *et al.* Oral iron acutely elevates bacterial growth in human serum. *Sci. Rep.* **5**, 16670; doi: 10.1038/srep16670 (2015).

## Figures and Tables

**Table 1:** Statistical analysis of *ex vivo* bacterial growth assays.

Analysis of Growth		*S. aureus*	*S. epidermidis*	*E. coli*	*S. Typhimurium*	*Y. enterocolitica*
Growth Curve	X^2^ (5 df)	6.05	65.70	225.00	232.00	117.70
	p-value	0.30	**<0.0001**	**<0.0001**	**<0.0001**	**<0.0001**
Time to Reach Peak Doubling Time (hours)	Mean pre (CI95%)	3.34 (3.21, 3.47)	13.7 (11.8, 15.7)	1.86 (0.50, 3.21)	Not Applicable - No MAX	Not Applicable - No MAX
	Mean post (CI95%)	3.25 (3.12, 3.38)	10.70 (10.0, 11.4)	2.56 (2.32, 2.79)		
	z	−1.26	−3.24	1.03		
	p-value	0.21	**0.001**	0.3		
Doubling Time During Exponential Phase (hours)	Mean pre (CI95%)	1.74 (1.67, 1.81)	4.82 (4.2, 5.5)	2.14 (1.99, 2.29)	2.03 (1.81, 2.26)	4.96 (3.0, 7.0)
	Mean post (CI95%)	1.75 (1.68, 1.82)	3.36 (3.0, 3.7)	1.50 (1.44, 1.56)	1.60 (1.37, 1.62)	4.37 (2.8, 6.0)
	z	0.28	−4.56	−8.04	−4.34	−1.73
	p-value	0.78	**<0.001**	**<0.0001**	**<0.001**	0.62

For each species of bacterium we compared its growth pre- and post-iron supplementation by examining differences in (1) Growth Curve, i.e. general pattern curve of the overall growth trajectories; (2) Time to Reach Peak Doubling Time, i.e. the time at which the rate of increase in log(OD_620_) was at a maximum; and (3) Doubling Time During Exponential Growth Phase.

**Table 2:** Statistical testing of the independent effects of iron supplementation and transferrin saturation (TSAT).

Hypothesis Testing		*S. aureus*	*S. epidermidis*	*E. coli*	*S. Typhimurium*	*Y. enterocolitica*
(1) Iron supp. affects growth	X^2^ (5 df)	5.92	55.70	221.00	213.00	108.17
	p-value	0.31	**<0.0001**	**<0.0001**	**<0.0001**	**<0.0001**
(2) Iron supp. affects growth independently of TSAT	X^2^ (5 df)	8.85	7.53	35.10	22.40	32.71
	p-value	0.12	0.18	**<0.0001**	**0.0004**	**<0.0001**
(3) TSAT affects growth	X^2^ (5 df)	9.79	55.50	300.00	348.00	120.41
	p-value	0.08	**<0.0001**	**<0.0001**	**<0.0001**	**<0.0001**
(4) TSAT affects growth independently of iron supplementation	X^2^ (5 df)	12.70	6.22	69.50	105.00	36.89
	p-value	0.03	0.29	**<0.0001**	**<0.0001**	**<0.0001**

Three models (mathematical equations) were fitted for each bacterial species: (a) iron supplementation × (t1 t2 t3 t4), i.e. iron supplementation, the time polynomials and their interactions; (b) TSAT × (t1 t2 t3 t4), i.e. TSAT, the time polynomials and their interactions; (c) (iron supplementation + TSAT) × (t1 t2 t3 t4), i.e. iron supplementation, TSAT and both their interactions with the time polynomials. We employed the likelihood ratio test to compare models for which the growth patterns were and were not dependent on TSAT or iron supplementation. The effects of iron supplementation and TSAT were derived from models (a) and (b) respectively and refer to the joint effects of the variable and the terms for its interaction with the time polynomials. The independent (conditional) effects of iron supplementation after controlling for TSAT, and TSAT after controlling for iron supplementation, are both derived from model (c) and again refer to the joint effects of the variable and the terms for its interaction with the time polynomials. For each bacterial species, each of the following four hypotheses were tested: (1) iron supplementation has an impact on bacterial growth; (2) iron supplementation has an impact on bacterial growth independently of TSAT; (3) TSAT has an impact on bacterial growth; and (4) TSAT has an impact on bacterial growth independently of iron supplementation. Significance was determined using a chi-squared test. Chi-squared and p-values are reported.
